# Molecular Mechanisms of Radiation-Induced Cancer Cell Death: A Primer

**DOI:** 10.3389/fcell.2020.00041

**Published:** 2020-02-13

**Authors:** Joseph Sia, Radoslaw Szmyd, Eric Hau, Harriet E. Gee

**Affiliations:** ^1^Department of Radiation Oncology, Peter MacCallum Cancer Centre, Melbourne, VIC, Australia; ^2^Division of Cancer Research, Peter MacCallum Cancer Centre, Melbourne, VIC, Australia; ^3^Sir Peter MacCallum Department of Oncology, The University of Melbourne, Melbourne, VIC, Australia; ^4^Children’s Medical Research Institute, Sydney, NSW, Australia; ^5^Sydney West Radiation Oncology Network, Sydney, NSW, Australia; ^6^The University of Sydney, Sydney, NSW, Australia

**Keywords:** radiotherapy, radiation therapy, stereotactic ablative radiotherapy, cell death, immunogenic cell death, abscopal effect

## Abstract

Radiation therapy (RT) is responsible for at least 40% of cancer cures, however treatment resistance remains a clinical problem. There have been recent advances in understanding the molecular mechanisms of radiation-induced cell death. The type of cell death after radiation depends on a number of factors including cell type, radiation dose and quality, oxygen tension, *TP53* status, DNA repair capacity, cell cycle phase at time of radiation exposure, and the microenvironment. Mitotic catastrophe (a pathway preceding cell death that happens in mitosis or as a consequence of aberrant mitotic progression) is the primary context of radiation-induced cell death in solid cancers, although in a small subset of cancers such as haematopoietic malignancies, radiation results in immediate interphase apoptosis, occurring within hours after exposure. There is intense therapeutic interest in using stereotactic ablative body radiotherapy (SABR), a precise, high-dose form of RT given in a small number of fractions, to prime the immune system for cancer cell killing, but the optimal radiation dose and fractionation remain unclear. Additionally, promising novel radiosensitisers targeting the cell cycle and DNA repair pathways are being trialled. In the context of the increasing use of SABR and such novel agents in the clinic, we provide an updated primer on the major types of radiation-induced cell death, focussing on their molecular mechanisms, factors affecting their initiation, and their implications on immunogenicity.

## Introduction

Radiation therapy (RT) is a major cancer treatment modality and is responsible for at least 40% of cancer cures (Ringborg et al., 2003), yet treatment resistance remains a clinical problem. A primary reason for this is the capacity for cancer cells to evade radiation-induced cell death. Treatment paradigms have traditionally viewed cancer as a cell-autonomous problem of dysregulated proliferation while side-lining host-tumour interactions, but this dogma has undergone a remarkable revolution in the last few decades, with increasing appreciation of the tumour stroma and immune milieu in shaping tumour evolution. Trials of novel radiosensitisers targeting not only key cell death pathways but also the stromal and immune microenvironment, particularly together with stereotactic ablative body radiotherapy (SABR), have gained intense interest.

Here, we provide an updated primer on the major types of radiation-induced cell death, focussing on their molecular mechanisms, factors affecting their initiation, and their implications on immunogenicity. We conclude with discussing these aspects in the context of the increasing use of SABR and novel agents in the clinic. We point readers interested in further detail to the excellent referenced reviews.

## The Cell Cycle and Types of Radiation-Induced Cell Death

The cell cycle is a highly regulated process occurring in two major phases: interphase (consisting of the G1, S, and G2 phases) and mitosis (cell division). During interphase, the cell grows its organelle counts (G1 phase), copies its DNA (S phase), and reorganises contents in preparation for division (G2 phase). Radiation-induced DNA damage is sensed by the kinases ataxia-telangiectasia mutated (ATM) and ataxia-telangiectasia and Rad3-related protein (ATR), which activate downstream proteins to initiate the DNA damage response (reviewed in Maier et al., 2016). This places the cell into cell cycle arrest, during which DNA double-strand breaks (DSBs) are repaired mainly by two pathways: non-homologous end joining (NHEJ) and homologous recombination (HR). NHEJ is error-prone but active throughout the cell cycle, while HR is error-free but requires an undamaged sister chromatid as the repair template, and therefore is only available in the late S and G2 phases. If damage is too significant for repair, cell death will occur via one of the types described below, at varying time points after the initial irradiation event (interphase or mitotic). In classical radiobiology, cell death is defined by loss of replicative capacity (i.e., replicative or reproductive death) and is determined by clonogenic assays. While this has undoubtedly served the field tremendously, it ignores the types and effects of cell death, discussed in this review.

### Mitotic Catastrophe and Mitotic Death

Mitotic catastrophe is a mechanism for the control of cells unable to complete mitosis, by the triggering of mitotic arrest and ultimately regulated cell death (RCD) or senescence. Mitotic death refers to RCD (usually intrinsic apoptosis) that is driven by mitotic catastrophe (Galluzzi et al., 2018). Because cells in mitotic catastrophe are almost always incapable of further replication, mitotic catastrophe is often confused as a bona fide cell death type. However, these cells eventually trigger one of the ‘other’ cell death pathways, or less commonly escape from this state (Gascoigne and Taylor, 2008; Vakifahmetoglu et al., 2008). Dysfunctional cell cycle checkpoints, a common hallmark of cancer cells, allow radiation-damaged cells to enter mitosis prematurely with misrepaired DNA, leading to mitotic catastrophe. The exact mechanisms for the initiation of mitotic catastrophe and the deciding of final cell fate are unclear. Several attempted divisions can occur before sufficient genetic damage is accumulated to trigger mitotic death, underlining why solid tumours often demonstrate delayed responses to RT.

### Apoptosis

Apoptosis is a highly regulated form of cell death with characteristic morphological and molecular features. The intrinsic (mitochondrial) apoptotic pathway is activated when the DNA damage repair machinery, with p53 as a central player, disrupts the balance between pro- and anti-apoptotic factors, resulting in the release of cytochrome c from the mitochondria into the cytoplasm to activate the intrinsic pathway-specific caspase 9 (Eriksson and Stigbrand, 2010). The extrinsic pathway, in contrast, is initiated by external signalling via binding of the tumour necrosis factor (TNF) family of ligands to plasma membrane death receptors, eventually causing downstream activation of the extrinsic pathway-specific caspase 8. Irradiated cells can upregulate death receptors, making them susceptible to death through this pathway (Wu et al., 1997; Chakraborty et al., 2003; Sheard et al., 2003). The third pathway, the ceramide pathway, is triggered by radiation-induced activation of acid sphingomyelinase in the plasma membrane, producing ceramide via hydrolysis of sphingomyelin. Radiation-induced DNA damage can also activate mitochondrial ceramide synthase for *de novo* synthesis of ceramide. Ceramide acts as a second messenger in initiating the apoptotic programme, but its signalling targets are complex (Pettus et al., 2002; Kolesnick and Fuks, 2003). The intrinsic, extrinsic and ceramide pathways converge in the activation of caspase 3 and 7, which sets off a cascade of controlled degradation of cellular components.

### Necrosis

Necrosis is an unregulated, chaotic form of cell death, triggered by unfavourable conditions such as extreme changes in pH, energy loss, and ion imbalance within the cell and its microenvironment after irradiation (Kroemer et al., 2009). Traditionally identified in histological specimens, it is a diagnosis of exclusion and based on morphology, characterised by a gain in cellular volume, plasma membrane rupture and spill of intracellular contents.

### Senescence

Senescence refers to permanent cell cycle arrest. Radiation-induced senescence is triggered by DNA damage and the induction of the p53 and pRb pathways causing cell cycle block, but other factors including oxidative stress may also be relevant triggers in the irradiation context (Sabin and Anderson, 2011; Li et al., 2018). Importantly, senescent cells while “dead” in terms of clonogenicity continue to be viable and metabolically active, over time developing a specific expression pattern of immunomodulatory factors. This senescence-associated secretory phenotype is a result of the extrusion of cytoplasmic chromatin fragments in senescent cells, which act on the DNA sensing and effector cGAS-STING pathway to drive expression of interferon and NF-kB elements (Dou et al., 2017; Gluck et al., 2017).

### Autophagy

Autophagy describes the process of sequestrating damaged or old cytoplasmic organelles within vesicles for lysosomal degradation in response to cellular stress. It is usually a cytoprotective process, but autophagic cell death occurs when the autophagic response is excessive (Kroemer and Levine, 2008). The autophagic machinery is a highly regulated pathway involving the ATG genes and is known to be triggered by irradiation, likely via the endoplasmic stress module and mTOR pathway, although the exact mechanisms are unclear (Tam et al., 2017). Autophagy observed after irradiation may be a mechanism of treatment resistance or of cell death, being likely context-dependent (Levy et al., 2017; Dikic and Elazar, 2018).

### Other Types of Cell Death

More recently, forms of RCD have been identified in response to irradiation, including necroptosis and ferroptosis, although the extent to which these occur *in vivo* are uncertain (Gong et al., 2019; Lang et al., 2019; Lei et al., 2019). Necroptosis has similar morphological appearances to necrosis, but is regulated. It can be activated by death receptors, but signalling is transduced via caspase-independent pathways converging on RIPK3 and MLKL. Ferroptosis is a type of cell death triggered by the accumulation of lipid peroxides as the lethal event due to decreased degradation by glutathione peroxidase (GPX4). With irradiation, this is shown to happen via ATM, which represses the cystene-glutamate antiporter system xc-, resulting in decreased glutathione, a co-factor for GPX4 (Lang et al., 2019).

## Factors Affecting Type of Cell Death

### Cell-Intrinsic Factors

The type of cell death after irradiation depends on a number of factors, with many remaining uncertainties. Cell type is a key determinant. In a subset of tumours such as haematopoietic malignancies (Radford et al., 1994; Jonathan et al., 1999), radiation results in immediate interphase apoptosis. In the majority of solid tumours however, mitotic catastrophe is the most frequent context of radiation-induced cell death (Eriksson and Stigbrand, 2010), whereas normal tissues commonly undergo senescence after irradiation (Nguyen et al., 2018).

The importance of cell type could be related to the cellular status and function of p53 and ATM. p53 is a key regulator of apoptosis and senescence. Intact p53 is required for interphase apoptosis (Fridman and Lowe, 2003), while disruptive TP53 mutations are associated with increased radioresistance via the inhibition of senescence (Skinner et al., 2012). As most tumour cells have lost normal p53 function or inactivated its downstream pathways, the G1/S checkpoint is impaired and DNA damage repair is consequently dependent on ATR/Chk1-mediated intra-S and G2/M arrest (Soussi and Beroud, 2001; Brady et al., 2011; Dillon et al., 2014). Thus, irradiated cells with inactivated p53 will enter mitosis with unrepaired DNA damage, resulting in mitotic catastrophe rather than immediate apoptosis or senescence (Ianzini et al., 2006). While manipulation of p21 or p53 levels may dramatically alter the kinetics of cell death after irradiation, it may not always correlate with the overall loss of replicative capacity (Wouters et al., 1997; Brown and Wouters, 1999). Similarly, ATM impacts cell death by regulating apoptosis, autophagy, and possibly necroptosis (Liang et al., 2013; Tripathi et al., 2013; Chen et al., 2015) (reviewed in Wu et al., 2017), but it is not clear yet how ATM may affect the decision between these types of cell death.

The phase of the cell cycle in which irradiation occurs also influences timing and type of cell death. Studies have demonstrated cells irradiated in G1 phase divide more times and survive longer before undergoing apoptosis compared to cells irradiated in G2 phase, while cells irradiated in mid-to-late S phase died without undergoing mitosis. Post-mitotic apoptosis however appeared to be stochastic and variable between cell lines (Forrester et al., 1999; Endlich et al., 2000).

### Radiation Factors

It may be tempting to speculate that ablative doses incline cells to a different type of cell death compared to conventional doses per fraction, but whether this occurs as a direct effect of irradiation is uncertain. The complexities of establishing this link surround dissecting the effect of dose per fraction from biological effective dose (BED), and accounting for temporal confounding by repopulation and cell death dynamics. Worth noting here is that the induction of sufficient damage to non-nuclear cellular components to evoke upfront necrosis may require clinically-irrelevant high doses (Warters et al., 1978). Nonetheless, the rate of apoptotic events after irradiation can be proportional to the number of fractions given, in line with the reassortment principle (cells cycling inter-fraction into states predisposed for interphase apoptosis) (Verheij and Bartelink, 2000).

Radiation quality may also affect mode of cell death. High linear energy transfer (LET) radiation such as alpha particles has been shown to result in enhanced chromosome rearrangements and reproductive death (Franken et al., 2011), due to both the complexity and absolute number of DNA damage clusters (Pinto et al., 2005; Obe et al., 2010).

### Microenvironment Factors

The above factors are strongly influenced by the cellular microenvironment. In brief, oxygen tension modulates cell death after irradiation, with a reduction in chromosomal aberrations and reproductive capacity of cells irradiated under hypoxic conditions. This is presumed due to kinetic competition between the oxygen ‘fixation’ of DNA damage and chemical repair processes (reviewed in Stewart et al., 2011). Under the hypoxic, nutrient-deprived conditions *in vivo*, cell death will be influenced both by the chemical properties of oxygen fixation and indirect effects of hypoxia on cellular processes including cell metabolism, DNA repair, and hypoxia inducible factor (HIF)-related survival mechanisms (Harris, 2002). An acidic microenvironment and serum deprivation also suppress the progression of cells to apoptosis and the formation of micronuclei after irradiation (Paglin et al., 1997; Park et al., 2000).

## Radiation-Induced Immunogenic Cell Death

The observation that an intact host immune system improves the tumour control probability of RT, first made in 1979 (Stone et al., 1979), suggests that the anti-tumour effects of RT must extend beyond its direct effects on cancer cells. Over the last decade, the concept of immunogenic cell death (ICD) has emerged to redefine cancer cell death from an immune-functional aspect, whereby the dying cell through peri-mortem processes is able to evoke anti-tumour adaptive immune responses (Ma et al., 2010; Kepp et al., 2014).

### Defining ICD

Historically, necrosis has been traditionally thought to be immunogenic (Scaffidi et al., 2002; Sancho et al., 2009), while apoptosis has been viewed as immunologically silent or even tolerogenic (Voll et al., 1997; Krysko and Vandenabeele, 2010). This relationship is an oversimplification. Apoptosis (Casares et al., 2005; Obeid et al., 2007b), necrosis (Scaffidi et al., 2002; Sancho et al., 2009), and autophagy (Michaud et al., 2011; Ko et al., 2014), have all been demonstrated to be capable of inducing immunogenic responses, while necrosis can in fact contribute to a chronic inflammatory microenvironment that is protumourigenic and immunosuppressive (Vakkila and Lotze, 2004).

The current framework for understanding ICD disengages from the traditional forms of cell death and requires two components: the exposure of danger signals, termed damage-associated molecular patterns (DAMPs), from the dying cell to alert antigen presenting cells (APCs), which include dendritic cells (DCs); and an antigenic epitope to be cross-presented by APCs for training of T cells (Galluzzi et al., 2017).

### Observations That RT Can Induce ICD

Bona fide ICD induced by RT has been directly demonstrated with *in vivo* vaccination assays in mouse models (Apetoh et al., 2007a; Obeid et al., 2007a; Gorin et al., 2014). A strong body of work now corroborate these landmark findings across different murine and human cell lines using surrogate measures (Deng et al., 2014; Gameiro et al., 2014; Golden et al., 2014; Ko et al., 2014; Lim et al., 2014). Radiation-induced ICD can also be implied from a growing number of pre-clinical and clinical studies combining RT with immunotherapy showing improved systemic tumour responses (reviewed in Deloch et al., 2016; Grassberger et al., 2019). Although the link is indirect, the release of DAMPs and antigenic determinants to evoke cancer-specific immunity in these studies are thought to be at least partially mediated through radiation-induced ICD. Other important trials testing RT for this role have yielded negative results for yet-uncertain reasons (Kwon et al., 2014; Voorwerk et al., 2019), suggesting that while the phenomenon of radiation-induced ICD is real, its desired clinical impact is a highly selective event.

### Mechanisms of Radiation-Induced ICD

Our understanding of the molecular mechanisms of radiation-induced ICD and the determinants for their efficient triggering are in its nascency. Calreticulin, HMGB1, and ATP, the classically described DAMPs, act on CD91, TLR4, and purinergic receptors on DCs respectively, to attract, activate, and promote phagocytosis of cellular corpses by DCs (Ma et al., 2010). Calreticulin, an endoplasmic reticulum chaperone, is translocated to the cell surface as part of the unfolded protein response in stressed cells as a pre-apoptotic event (Obeid et al., 2007b). HMGB1 is a nuclear chromatin-binding protein that is released on necrosis (Scaffidi et al., 2002; Apetoh et al., 2007b). Unlike HMGB1 where passive release can occur, optimal secretion of ATP in radiation-induced ICD is active and dependent on intact pre-mortem autophagic machinery for the accumulation of ATP within autolysosomes (Michaud et al., 2011; Ko et al., 2014). A host of other DAMPs have since been identified, including cytosolic double stranded DNA (dsDNA), the heat shock proteins 70 kDa (HSP70) and 90 kDa (HSP90), F-actin, and interleukin-33 (reviewed in Krysko et al., 2012; Galluzzi et al., 2017).

Cytosolic dsDNA and the cGAS-STING axis have recently emerged as subjects of intense therapeutic interest. dsDNA is released from either the mitochondria and/or nucleus of irradiated cells into the cytoplasm and binds to its sensor cGAS, producing the cyclic dinucleotide cGAMP. Micronuclei that accumulate when irradiated cells progress through mitosis with radiation-induced DSBs also constitute a source of dsDNA (Harding et al., 2017). cGAMP ligates with the adaptor protein STING to ultimately upregulate the expression of type I interferon, an important immunomodulatory cytokine (Burdette and Vance, 2013; Paludan and Bowie, 2013). Importantly, dsDNA does not need to activate STING in a cell-intrinsic fashion, but dsDNA or cGAMP can be released upon ICD and taken up by myeloid cells, including DCs, to activate STING within those cells (Deng et al., 2014; Diamond et al., 2018; Schadt et al., 2019). This axis can be shut down by ablative radiation doses due to the simultaneous induction of the exonuclease TREX1, which degrades cytosolic dsDNA (Vanpouille-Box et al., 2017). While this mechanism explains the negative impact of ablative radiation doses on abscopal responses in a murine mammary carcinoma model (Dewan et al., 2009), the observation is not consistently held up in non-abscopal readouts of tumour immunity, suggesting that other factors may be involved in this regulation (Burnette et al., 2011; Deng et al., 2014; Schadt et al., 2019). On the other hand, generation of DAMPs such as ATP and HMGB1 appears to be proportional to radiation doses of up to 100 Gy *in vitro* (Gameiro et al., 2014), but whether this translates to increased immunogenicity *in vivo* is unknown.

On the antigenicity side, RT increases the processing and MHC-I-restricted presentation of surface antigens in a cell-autonomous manner, at least partially via the mTOR pathway (Santin et al., 1997; Verbrugge et al., 2014). More provocatively, as a result of altered transcriptional activity and antigen processing, irradiated tumour cells *in vitro* demonstrate a diversification of epitope repertoire presented on MHC-I (Reits et al., 2006). A recent clinical trial combining RT with immune checkpoint blockade reported a patient case with expansion of T cell clones against a radiation-induced neoantigen, serving as the first in-human proof of this concept (Formenti et al., 2018).

## Discussion and Clinical Relevance

**FIGURE 1:**
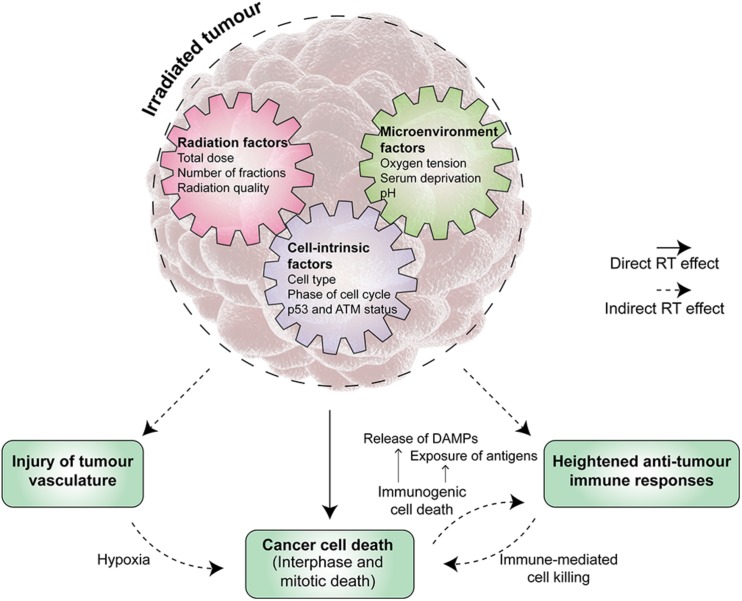
The anti-tumour activity of radiation therapy (RT) is multi-faceted. Tumour irradiation induces both direct and indirect effects in controlling the tumour. Direct effects are the result of significant radiation-induced DNA damage in cancer cells leading to their death, which may occur at various time points after the initial irradiation event. Radiation therapy (RT) also exerts anti-tumour activity via indirect effects, which include, but are not limited to, injury of tumour vasculature and priming of host anti-tumour immune responses. A range of factors influence the triggering and magnitude of these effects, broadly falling into cell-intrinsic, radiation, and microenvironment factors. A deeper knowledge of the underpinning mechanisms and their interplay will reveal opportunities for enhancing the overall anti-tumour activity of RT. ATM, ataxia-telangiectasia mutated. DAMP, danger associated-molecular pattern.

Increasingly, our appreciation of the anti-tumour effects of RT extends beyond a DNA and cancer cell-centric perspective (Figure 1). The need to better understand the molecular pathways of radiation-induced cell death is even more important in light of recent technological advances in the safe delivery of RT and the increasing use of novel agents in clinic. SABR is progressively adopted into routine clinical practice with a growing number of studies backing its remarkable efficacy, greater than predicted by a simple extrapolation from lower doses per fraction. For example, it has been shown to double median survival for oligometastatic disease (Palma et al., 2019) and more than double the response rate to immunotherapy (Formenti et al., 2018; Theelen et al., 2019), leading to the hypothesis that the biology of tumour response to irradiation is different when given high dose per fraction. Theoretically, SABR would induce DNA damage that was more difficult to repair and decrease tumour cell repopulation due to reduced overall treatment time, albeit with a cost from decreased inter-fraction cellular reassortment and reoxygenation. There is currently insufficient data to confidently propose a model by which SABR achieves such high efficacy (Moding et al., 2016; Song et al., 2019). Indeed, some authors argue that the improved local control seen with SABR purely results from a higher dose (Brown et al., 2013, 2014).

Others posit that SABR has additional indirect effects on cancer cells. Significant tumour vascular injury has been shown to result from SABR, leading to hypoxic cancer cell death several days after irradiation (Song et al., 2019). Interestingly, the ceramide apoptotic pathway in the tumour vascular endothelium, activated with ablative radiation doses, is thought to be especially important (Garcia-Barros et al., 2003; Rao et al., 2014). These results, however, have been directly challenged in other models (Moding et al., 2015), while others contend that hypoxia induced by SABR in fact inhibits its efficacy (Carlson et al., 2011).

The other reason measurements of radiation-induced cancer cell death *in vitro* fail to represent the extent of clinically observed responses is due to host immune responses (Golden et al., 2012; Haikerwal et al., 2015). For example, inhibition of the pro-survival autophagy machinery radiosensitises cell kill *in vitro*, but in fact impairs radiation tumour control in immunocompetent mice due to loss of autophagy-dependent ATP release as an immune adjuvant (Ko et al., 2014). As such, a pressing question is the impact of radiation dose-fractionation on cell death pathways, especially those related to ICD. Studying fractionated regimens in the pre-clinical setting is complicated because of the physiologically artificial context and logistical difficulties of irradiating animals *in vivo* over an extended number of fractions. Pre-clinical studies therefore typically employ moderately hypofractionated or ablative regimens (Demaria and Formenti, 2012; Deloch et al., 2016), potentially biassing the commonly-held hypothesis that such regimens are better inducers of ICD.

**DEFINITIONS**

**Radiation therapy terms:**

**Fraction** – single treatment session into which the overall radiation therapy course is broken down.

**Stereotactic ablative body radiotherapy/stereotactic body radiation therapy (SABR/SBRT)** – precise, high-dose radiation therapy given in a small number of fractions.

**Abscopal effect** – regression of a tumour lesion distant to the radiation field.

**Oligometastatic** – Stage IV (distant spread) but with a limited number of metastases (usually less than five).

**Linear energy transfer (LET)** – measure of radiation quality, describing the pattern of energy deposition by the radiation.

**Biological effective dose (BED)** – an expression of total dose for comparison of biological effects between radiation dose-fractionation regimens.

**Reassortment** – the redistribution of cells previously in radioresistant phases into more radiosensitive phases after each radiation fraction.

**Oxygen fixation** – the making permanent of DNA damage induced by radiation, via the formation of irreparable peroxyl radicals.

Finally, greater understanding of the mechanisms of cell death will enable the application of novel radiosensitisers (Moding et al., 2016). For example, the dependence of many cancer cells specifically on the G2/M checkpoint has led to the development of agents that target this checkpoint, particularly Chk1, Wee1, ATM, and ATR (Dillon et al., 2014). Hypoxia-modifying therapy might prevent HIF-mediated revascularisation, recurrence, and metastasis (Moding et al., 2016). Additionally, targeting previously under-appreciated cell death types such as ferroptosis may support the combination of RT and immunotherapy (Lang et al., 2019). Alongside these developments, biomarker driven studies must not be neglected to help guide patient selection.

## Conclusion

Cancer cells undergo a range of interphase and mitotic death after irradiation via direct and indirect effects of RT. An increased understanding of the molecular mechanisms of radiation-induced cell death will reveal novel opportunities for improving the overall anti-tumour efficacy of RT.

## Author Contributions

JS, RS, EH, and HG conceived and designed the study, revised the manuscript, and read and approved the submitted version. JS wrote the first draft of the manuscript. HG wrote sections of the manuscript.

## Conflict of Interest

The authors declare that the research was conducted in the absence of any commercial or financial relationships that could be construed as a potential conflict of interest.
